# Supplementary material for: Identification of periodic attractors in Boolean networks using a priori information
Source: PLoS Comput Biol. 2022 Jan 14;18(1):e1009702. doi: 10.1371/journal.pcbi.1009702 (PMC8803189; doi:10.1371/journal.pcbi.1009702)
Supplement: S1 File — Fig A. Attractors of typical and atypical behavior inducing microenvironments. In the original study [9], the authors identified 35 microenvironments which induce typical endothelial cell (EC) behavior, and 32 microenvironments which induce atypical EC, behavior using a reduced network with n = 64 nodes. We show the types of attractors (point, periodic, and lengths of periodic attractors) from our analysis of the full network with n = 142 nodes. As initial guesses, the previously defined values for each microenvironment comprising 16 nodes were applied. Additionally, 10 nodes with a fixed value were used. For the remaining 116 nodes, two scenarios were tested, one in which all remaining nodes are set to 1 (ON scenario), and one where all remaining nodes are set to 0 (OFF scenario). As a priori information, the probabilities 0,7, 0.8, 0.9, and 1 were tested. Hence, for each microenvironment and ON or OFF setting, four tests were performed. For each number and scenario, the same results were retrieved (exception for 1). The results for a priori probability 0.7 are shown. Left: Attractor distributions for microenvironments (numbers 1–35) inducing typical behavior. Right: Attractor distributions for microenvironments inducing atypical (numbers 36–67) behavior. Fig B. Endothelial cell (EC) behavior of atypical behavior inducing microenvironments. In the original study [9], the authors identified 32 microenvironments predicted to induce atypical EC behavior. EC behavior is interpreted by the signature of four markers (AKT1, autocrine JAG1, DLL4a, and NRP1). A microenvironment induces atypical EC behavior if the EC marker signature does not correspond to phalanx, stalk, or tip, or if their signature is not stable in the detected attractors. We based our initial guesses required for attractor analysis on the information provided in the original study, from where we can assign node values for 16 nodes comprising the microenvironment, and 10 nodes with fixed values. For the r [file pcbi.1009702.s001.pdf]

Supplementary Information for Identification of periodic attractors in Boolean networks using *a priori* information

Ulrike Münzner[1,2], Tomoya Mori[2], Marcus Krantz[3], Edda Klipp[3], Tatsuya Akutsu[2]

January 25, 2022

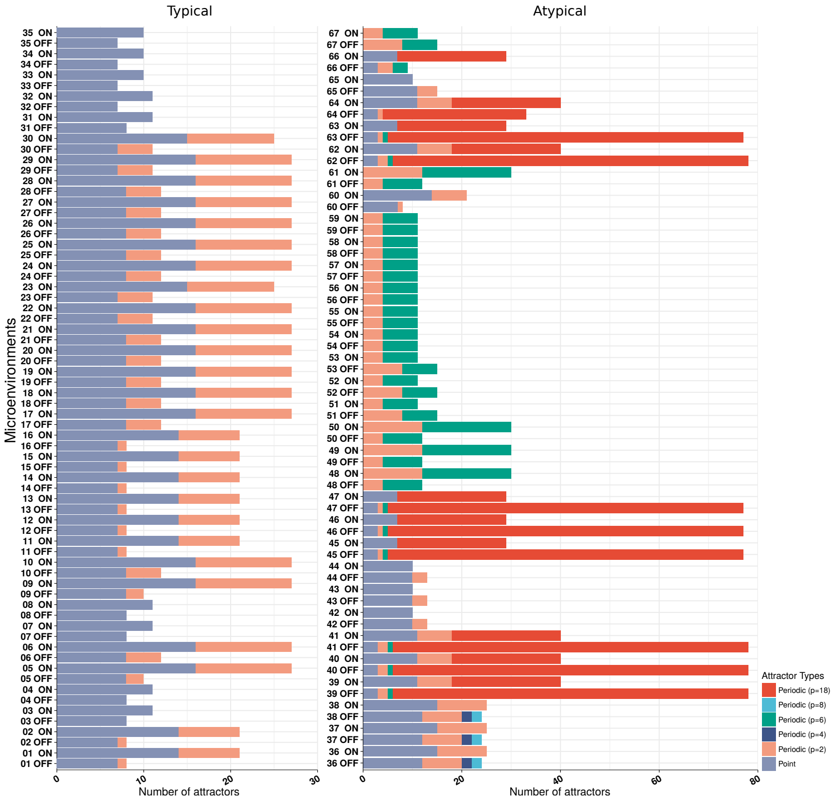

Figure A. **Attractors of typical and atypical behavior inducing microenvironments.** In the original study [3], the authors identified 35 microenvironments which induce typical endothelial cell (EC) behavior, and 32 microenvironments which induce atypical EC, behavior using a reduced network with $n = 64$ nodes. We show the types of attractors (point, periodic, and lengths of periodic attractors) from our analysis of the full network with $n = 142$ nodes. As initial guesses, the previously defined values for each microenvironment comprising 16 nodes were applied. Additionally, 10 nodes with a fixed value were used. For the remaining 116 nodes, two scenarios were tested, one in which all remaining nodes are set to 1 (ON scenario), and one where all remaining nodes are set to 0 (OFF scenario). As *a priori* information, the probabilities 0,7, 0.8, 0.9, and 1 were tested. Hence, for each microenvironment and ON or OFF setting, four tests were performed. For each number and scenario, the same results were retrieved (exception for 1). The results for *a priori* probability 0.7 are shown. *Left:* Attractor distributions for microenvironments (numbers 1-35) inducing typical behavior. *Right:* Attractor distributions for microenvironments inducing atypical (numbers 36-67) behavior.

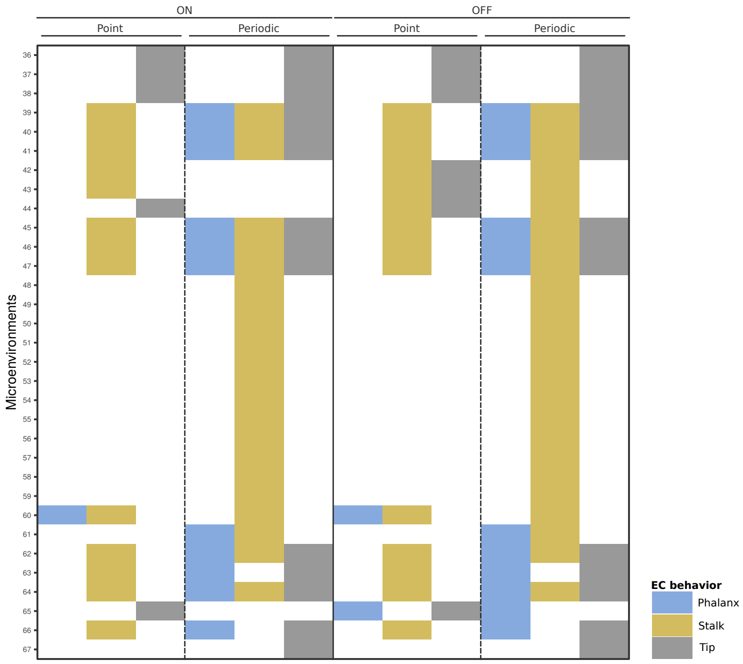

Figure B. Endothelial cell (EC) behavior of atypical behavior inducing microenvironments. In the original study [3], the authors identified 32 microenvironments predicted to induce atypical EC behavior. EC behavior is interpreted by the signature of four markers (*AKT1*, autocrine *JAG1*, *DLL4a*, and *NRP1*). A microenvironment induces atypical EC behavior if the EC marker signature does not correspond to phalanx, stalk, or tip, or if their signature is not stable in the detected attractors. We based our initial guesses required for attractor analysis on the information provided in the original study, from where we can assign node values for 16 nodes comprising the microenvironment, and 10 nodes with fixed values. For the remaining 116 out of 142 nodes, we tested two scenarios, one in which all remaining nodes are set to 1 (ON scenario), and one where all remaining nodes are set to 0 (OFF scenario). Shown are the EC behaviors interpreted from the detected attractors from our analysis. Rows: Microenvironment numbers (numbers 36-67 corresponding to the original numbering) predicted to induce atypical EC behavior. *ON* column: Results from scenarios where the unkown node values in the initial guess were set to 1 (ON). *OFF* column: Results from scenarios where the unkown node values in the initial guess were set to 0 (OFF). Rows (microenvironments) which resulted in attractors corresponding to only one EC behavior (numbers 36-38, and 48-59) showed an instable EC marker signature, and are regarded to induce atypical behavior.

A

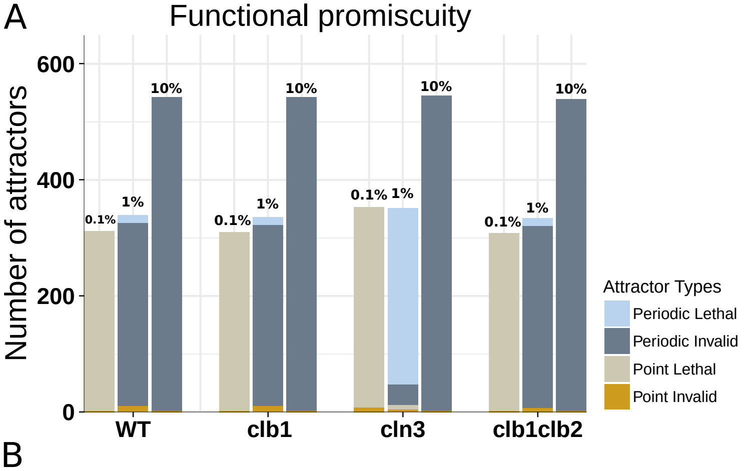

B

Type	WT			clb1			cln3			clb1clb2		
Rate	0.1 %	1%	10%	0.1%	1%	10%	0.1 %	1 %	10%	0.1%	1%	10%
Per. Lethal	0	13	0	0	13	0	0	304	0	0	13	0
Per. Inval.	0	315	540	0	312	540	0	36	543	0	314	537
Point Lethal	310	0	0	308	0	0	345	7	0	306	0	0
Point Inval.	10	11	2	2	11	2	8	5	2	2	7	2

Figure C. Attractors of functionally promiscuous mutants. Results from the attractor analysis of a cell cycle control network with $n = 3158$ nodes. The Boolean rules in the original network were mutated from AND to OR, mimicking a functionally promiscuous mutant. Three networks were generated with mutation rates of 0.1%, 1%, 10%. For each network, four initial guesses were used, the wildtype genotype, and the *clb1*, *cln3*, and *clb1clb2* mutant genotypes. The detected attractors were interpreted in terms of validity, and viability of the corresponding phenotype. Due to the bipartite Boolean modeling formalism, our proposed algorithm may detect attractors where two or more mutually exclusive nodes are active, or essential components are inactive. While these types of attactors are technically possible to detect, they do not carry biological meaning and we refer to them as *invalid*. The remaining attractors are regarded as biologically valid attractors, with two possible corresponding phenotypes: *viable* and *lethal*. (A) Detected attractors using four initial guesses, and three mutational rates which affect the Boolean rules. (B) Data table. WT: wildtype, Per.: Periodic, Inval.: Invalid.

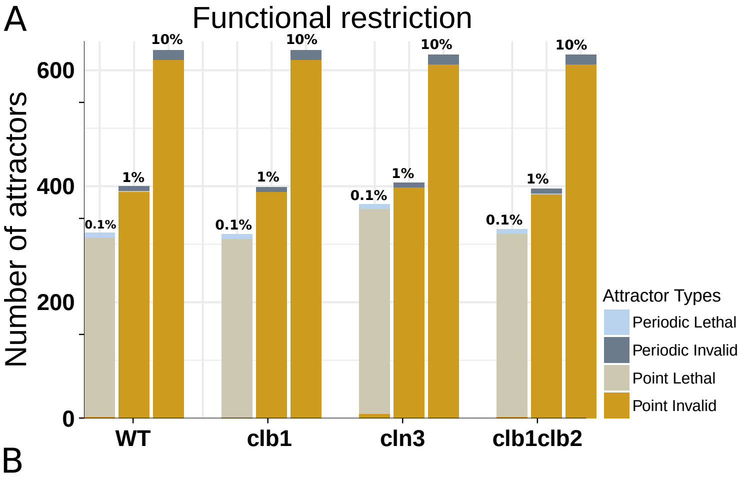

B

Type	WT			clb1			cln3			clb1clb2		
Rate	0.1 %	1%	10%	0.1%	1%	10%	0.1 %	1 %	10%	0.1%	1%	10%
Per. Lethal	8	0	0	8	0	0	8	0	0	8	0	0
Per. Inval.	0	8	17	0	8	17	0	8	17	0	8	17
Point Lethal	310	2	0	307	0	0	353	0	0	316	2	0
Point Inval.	2	390	618	2	390	618	8	398	610	2	386	610

Figure D. **Attractors of functionally restricted mutants.** Results from the attractor analysis of a cell cycle control network with $n = 3158$ nodes. The Boolean rules in the original network were mutated from OR to AND, mimicking a functionally restricted mutant. Three networks were generated with mutation rates of 0.1%, 1%, 10%. For each network, four initial guesses were used, the wildtype genotype, and the *clb1*, *cln3*, and *clb1clb2* mutant genotypes. The detected attractors were interpreted in terms of validity, and viability of the corresponding phenotype. Due to the bipartite Boolean modeling formalism, our proposed algorithm may detect attractors where two or more mutually exclusive nodes are active, or essential components are inactive. While these types of attactors are technically possible to detect, they do not carry biological meaning and we refer to them as *invalid*. The remaining attractors are regarded as biologically valid attractors, with two possible corresponding phenotypes: *viable* and *lethal*. (A) Detected attractors using four initial guesses, and three mutational rates which affect the Boolean rules. (B) Data table. WT: wildtype, Per.: Periodic, Inval.: Invalid.

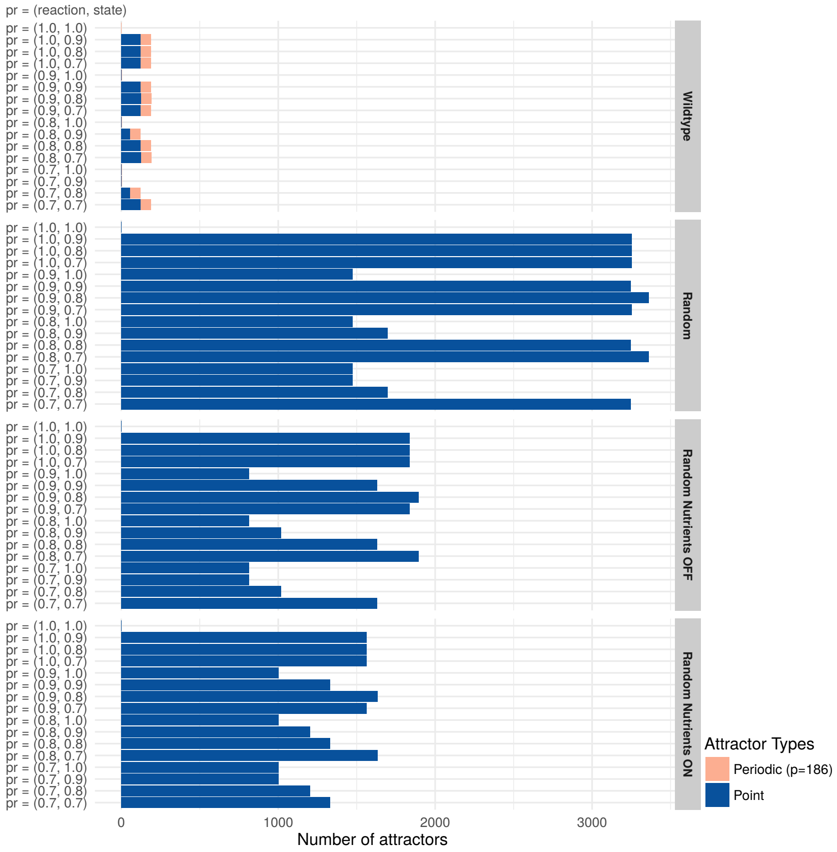

Figure E. **Attractors using either a known periodic attractor or random initial states in the cell cycle control network.** Results from the attractor analysis of a cell cycle control network with $n = 3158$ nodes using either the wildtype genotype as initial guess (upper panel), or in total 10 random initial guesses (second panel from above). Additionally, these 10 random initial guesses were in one scenario modified so that the network input *Nutrients* was set to 0 (OFF scenario, third panel from above), and in another scenario so that the network input *Nutrients* was set to 1 (ON scenario, bottom panel). Furthermore, in the bipartite network, there are nodes corresponding to biochemical reactions, and nodes corrsponding to elemental states of the components included, resulting in two node types. For each node type, the same *a priori* probability was used (0.7, 0.8, 0.9, or 1.0), and the pairwise combinations of the *a priori* probabilities between the two node types tested. The y-axis labels indicate the *a priori* probabilities for reaction and state nodes. Results shown for random initial state 1, respectively. p: period; pr: probability.

Table A. Periodic attractors and their corresponding phenotypes using wildtype as initial guess (related to Fig. 4, main manuscript). *Viable:* correctly identified viable genotypes; *not testable:* cellular function where corresponding gene product not identified. Parenthesis: Number of attractors. Not shown: wildtype attractor (1), technical (7), *sec2* (1, lethal), *net1*, *pp2a* (2, contradictory). *Technical:* initial conditions included none or more than one of the mutual exclusive cell cycle stages; *lethal:* incorrectly identified as viable, true phenotype lethal; *contradictory:* contradictory statements reported in literature. All found attractors are unique. Viability status from The Saccharomyces Genome Database (SGD) [1].

	Viable (39)	Not testable (16)
1	*ace2*	cdc10ppt
2	*ase1*	cdc14ppt
3	*bem2*	cdc24ppt
4	*bem3*	cdc28ppt
5	*bfa1*	cdc3ppt
6	*bmh1*	chs2ppt
7	*bmh2*	dbf2ppt
8	*bnr1*	hof1ppt
9	*boi1*	hsl7ppt
10	*boi2*	kin4kinase
11	*bud2*	lte1ppt
12	*chk1*	nrm1ppt
13	*cin8*	nud1ppt
14	*clb1*	pds1ppt
15	*clb2*	rad53ppt
16	*clb3*	spc110ppt
17	*clb4*	
18	*far1*	
19	*fin1*	
20	*fkh1*	
21	*inp51*	
22	*lrg1*	
23	*lte1*	
24	*mad3*	
25	*mih1*	
26	*ptc2*	
27	*ptc3*	
28	*rga1*	
29	*rga2*	
30	*sac1*	
31	*sgs1*	
32	*slk19*	
33	*ssa1*	
34	*stb1*	
35	*swi5*	
36	*tgl4*	
37	*whi3*	
38	*ydj1*	
39	*yhp1*	

Table B. **Point attractors and their corresponding phenotypes using wildtype as initial guess (related to Fig. 4, main manuscript).** *Essential:* Correctly identified as lethal mutants; *non-essential:* incorrectly identified as lethal, paralogs with compensating function *in vivo* not included in model; *structural:* nodes in network corresponding to structural compounds (e.g. polymerase II) not modelled as individual gene products or corresponding to a cellular state (e.g. ssDNA); *not testable:* cellular function where corresponding gene product not identified, *(*)* contradictory statements reported in the literature. Parenthesis: Number of detected attractors. Double mutants with *cdh1* were found for attractors occurring twice. Not shown: *technical* where initial conditions included none or more than one of the mutual exclusive cell cycle stages (2); attractors where genotype could not be identified (2). All found attractors are unique. Viability status from SGD [1].

	Essential	Non-essential	Structural	Not testable
1	*cak1* (2)	*bem1* (2)	APC (2)	ndd1ppt (2)
2	*cdc11(*)* (2)	*bni1* (2)	Decay (2)	swe1ppt (2)
3	*cdc12* (2)	*cdh1* (4)	Polymerase II (2)	
4	*cdc14* (2)	*cdh1 + clb1* (1)	Proteasome (2)	
5	*cdc20* (2)	*cdh1 + clb2* (1)	RFC (2)	
6	*cdc24* (2)	*cla4* (2)	Ribosome (4)	
7	*cdc28* (2)	*cnm67* (2)	ssDNA (2)	
8	*cdc45* (2)	*dun1* (2)		
9	*cdc5* (2)	*fkh2gene* (2)		
10	*cdt1* (2)	*hsl7* (2)		
11	*dpb11* (2)	*mrc1* (2)		
12	*mcm1* (2)	*sml1* (2)		
13	*mec1* (2)	*spc72* (2)		
14	*mss4* (2)	*swi6* (2)		
15	*ndd1gene* (2)			
16	*nud1* (2)			
17	*orc1* (2)			
18	*orc2* (2)			
19	*orc6* (2)			
20	*rfa1* (2)			
21	*rfa2* (2)			
22	*rho1* (2)			
23	*scc2(*)* (2)			
24	*scc3* (2)			
25	*scp97* (2)			
26	*sfi1* (2)			
27	*sld2* (2)			
28	*sld3* (2)			
29	*smc1* (2)			
30	*smc3* (2)			
31	*smc4* (2)			
32	*spc110* (2)			
33	*spc29* (2)			
34	*spc98* (2)			
35	*stt4(*)* (2)			
36	*tub1* (1)			
37	*tub4* (2)			

References

[1] J. M. Cherry, E. L. Hong, C. Amundsen, R. Balakrishnan, G. Binkley, E. T. Chan, K. R. Christie, M. C. Costanzo, S. S. Dwight, S. R. Engel, D. G. Fisk, J. E. Hirschman, B. C. Hitz, K. Karra, C. J. Krieger, S. R. Miyasato, R. S. Nash, J. Park, M. S. Skrzypek, M. Simison, S. Weng, and E. D. Wong, "Saccharomyces Genome Database: the genomics resource of budding yeast," *Nucleic Acids Res.*, vol. 40, no. D1, pp. D700–D705, Nov. 2011.

[2] U. Münzner, E. Klipp, and M. Krantz, "A comprehensive, mechanistically detailed, and executable model of the cell division cycle in saccharomyces cerevisiae," *Nat. Comm.*, vol. 10, no. 1, p. 1308, 2019.

[3] N. Weinstein, L. Mendoza, I. Gitler, and J. Klapp, "A network model to explore the effect of the micro-environment on endothelial cell behavior during angiogenesis," *Frontiers in Physiology*, vol. 8, p. 960, 2017.
