# Supplementary material for: Identification of periodic attractors in Boolean networks using a priori information
Source: PLoS Comput Biol. 2022 Jan 14;18(1):e1009702. doi: 10.1371/journal.pcbi.1009702 (PMC8803189; doi:10.1371/journal.pcbi.1009702)
Supplement: Attachment — Submitted filename: longattresponse_merged.pdf [file pcbi.1009702.s004.pdf]

Response Letter

Dear Editors and Reviewers,

We appreciate your comments on our manuscript. We revised the manuscript taking all your comments into account.

In the revision process, we found minor errors in Lemma 1 and Theorem 1: polynomial factors in the expected number of trials should be n^2 instead of n. Therefore, we corrected the statements of Lemma 1 and Theorem 1 and their proofs. We also corrected some other parts depending on this factor. Please note that polynomial factors are much smaller than exponential factors for non-small n and thus we believe that this modification does not detract the significance of our obtained theoretical results.

Our responses to the comments of the reviewers are detailed in the following pages. The page/paragraph numbers in the replies refer to the new manuscript unless otherwise stated.

Additionally, we amended technical issues: We added legends and information about the three supplementary files S1-3 to the manuscript, and updated supplementary files S2-3. We also added a Data Availability statement and a Funding statement to the manuscript.

Sincerely,

Tatsuya Akutsu (Corresponding Author)
Kyoto University

1 Response to Reviewer 1

Comment. Reading your paper on the development of efficient detection of cyclic attractors in large Boolean networks based on a priori information about the expected state of a subset of nodes convinced me that your approach is innovative and potentially quite useful, as we as a field scale up to Boolean models of increasing size and incredibly unmanageable attractor repertoire. Thus, most of my comments below are ideas on how to articulate the merit of this method more clearly. I believe improving the clarity of the paper and describing the need and applications for your method more compelling would significantly improve your manuscript.

Response. We thank you for your valuable comments. We revised the manuscript according to your comments as detailed below.

Comment. 1. Articulating your motivation: – I appreciated the introduction articulating WHY the size of the state space of potential initial conditions, 2^n, is a problem for large networks. However, instead of considerable effort to convince readers that a speedup is a good thing, I think it would be important to explain at the start WHY an algorithm a priori information about the desired state of some or all nodes is GENERALLY or very often useful.

Response. As for the importance of breaking the 2^n barrier, we have added more sentences in the second and third paragraphs of the Introduction (for details, please see Response to Reviewer 3). The importance of use of a *priori information* is explained in the response to the next comment, and in the last paragraph of the Introduction.

Some questions to consider here: * When do we have this type of a priori information? Biological examples above and beyond 2 already built models would help

Response. Thank you for helping us to clarify this important point of our manuscript. We now added the following explanation about *a priori* information:

> The *a priori* information reflects the expected state (0 or 1) of each node and the confidence in this state (a probability $0 \leq p \leq 1$). The first information can be inferred from binarization of experimental data, *e.g.* by assigning the value 1 to an expressed gene. We refer to this information as initial guess. The second part, the probability, is much more difficult to infer from experimental data. However, the beauty of the method is that this value can be tried out empirically: The higher the confidence, the later the value will be switched by the algorithm. Of course, there are also situations when the value can be inferred from data: *E.g.*, the gene can be detected (over a certain threshold) in 7 out of 10 time points: $p_{true} = 0.70$.

* How general is the situation in which your method could aid modelers examine their models in more depth than before?

Response. We show how to analyze two Boolean models with different scope, size, and modeling formalism and expect that our algorithm can be applied to a variety of Boolean models. Since smaller Boolean models can be analyzed with existing algorithms, we set out to demonstrate the usefulness of our algorithm to larger Boolean networks with non-linear behavior. We added the following motivation why we chose the two examples:

> The authors deemed the original network with $n = 142$ nodes too large for attractor analysis, hence, the attractors of the full network remain unknown. This makes the full network an excellent target to demonstrate that our proposed algorithm can easily be applied to a network of this size. Furthermore, our analysis enables us to compare the results from two networks which have been proposed to contain the same information.

And:

> To the knowledge of the authors, a biological bBM of the size of the cell cycle control network has not yet been analyzed using a semi-automatic approach to study the attractor landscape. Hence, the cell cycle control network is a good example to demonstrate the feasibility of our proposed algorithm to the analysis of large-scale BNs, as well as to explore if attractors other than the known ones exist.

We are not aware of any other larger or more challenging Boolean models to test. We consider our algorithm to be useful for any Boolean model that is too large for an exhaustive attractor search.

Comment. – In the Discussion, the sentence "In addition, a priori information might be useful to efficiently stabilize biological systems" should be explained in 1-2 sentences; just noting that this is the case without context (requiring a side-trip to the citation) is not that helpful; it leaves readers wondering: why? how? when?

Response. We have modified this sentence as below.

> In addition, *a priori* information might be useful to efficiently stabilize biological systems [12, 13], where a BN is called *stabilizable* if there exists a feedback control and a point attractor such that the BN can be driven to the attractor beginning from any state. *A priori* information might be utilized to determine such a point attractor.

Comment. 2. Clarifying the logic of your pseudo-code:
– I think that describing the heart of your algorithm in 1-2 sentences before introducing the pseudo-code would make reading this part of the paper much smoother. I would recommend clarifying that your method start testing for attractors from the "best" subspace of $x0 \in \{0, 1\}^n$, one in which all nodes match the a priori "known" final attractor pattern. If the search from this subspace fails, the method moves away from these "most expected patterns" by increasing the number of bits that don't match, one at a time. There are likely clearer ways to phrase this, but a short explanation goes a long way in helping people read the pseudocode.

Response. We have added the following sentences.

> It starts testing for attractors from the most plausible vector $\mathbf{x}_0 \in \{0, 1\}^n$ with following the trajectory starting from it. If the search from this vector fails, the algorithm examines trajectories from the vectors each of which has one bit different from the first one. It further repeats the same procedure by increasing the number of bits different from the first vector.

We have also added the following sentence at the end of the "Algorithm" Subsection.

> In this version, at most 3 different values can be specified as probabilities (including probability 1.0) and bit vectors are examined from higher probabilities one to lower ones under a constraint that the number of changed bits for each probability class must not exceed the specified threshold.

Comment. – Most important: please explain / clearly define what a "desired" attractor is! Since your algorithm does more than simply verify the existence of a cycle that goes through a specific single network state - what, exactly is the definition of a desired attractor that stops the search?

Response. Desired attractors depend on users and cannot be mathematically defined. Although we gave some discussions on desired attractors in the last part of "Time complexity analysis" Section, we have newly added the following sentences just below the pseudocode.

> Note that how to decide whether the current attractor is a desired one is not a trivial task. If we know some exact criteria (e.g., allowable range of the attractor period and/or states of specific nodes), we can add a subroutine to check it. Otherwise, the algorithm can be terminated if the number of trials exceeds a specified number or CPU time exceeds the time limit. Then, we may manually check (maybe with some user-customized computer program to rule out obviously non-desired ones) whether or not there exists a desired one among the attractors reported so far.

For Lemma 1 and Theorem 1, we need to give a precise definition of the desired attractor (precisely, the target state). Therefore, we have added the following sentences.

> Suppose that $x_i = 0$ holds with probability p for all $i = 1, \ldots, n$ in some specific global state $\mathbf{x}_t$ of the target attractor, where $p > 0.5$. Note that if $x_i = 1$ has the probability p ($p > 0.5$) it is enough to exchange the roles of 0 and 1 for such nodes. In theoretical analyses, the objective is to give an upper bound of the number of trials until the algorithm examines $\mathbf{x}_t$ as a starting vector. Therefore, we need to modify STEP 3 as follows:
>
> > If $\mathbf{x}_0 = \mathbf{x}_t$, output it and exit.
>
> Of course, this modified algorithm is meaningless in practice because it is impossible to know $\mathbf{x}_t$ in advance. However, the expected number of trials (i.e., the expected number of tested $\mathbf{x}_0$s) for this modified algorithm gives an upper bound of that for the original algorithm because it examines the whole trajectory beginning from $\mathbf{x}_0$ (much more than one global state $\mathbf{x}_0$ per trial).

Comment. 3. Making the mathematics more accessible: – This comment reflects the bias of someone who can follow your math but has to work quite hard to figure out what you meant to do first, and then check if this is indeed what your formulas do. I recommend a beefing up of the math section with more text, and targeting a broader audience to better match the readership of Plos Comp Bio. I am thinking of readers who do not routinely approach problems in a

mathematical fashion, and thus they can't read your intentions off from your formulas, but who could nevertheless follow your logic if guided. To this end, I recommend adding explanations that set up what each step / formula is designed to accomplish, and THEN showing the formula itself. For example : "First, we evaluate the partial sum F (n, p) of E (n, p) defined by ..." * Explain why you evaluate these sums. Explain what is it that Fn counts and were does each of the terms in the sum come from. Think of it as explaining it to a student, since your audience is likely looking for a quick understanding of your logic rather than a math puzzle. – I recommend a similar introduction to every step in your proofs. These manipulations quickly become trivial with use, but can represent a steep initial barrier to a large swat of your potential readers.

Response. We have added many explanations in the proofs of Lemma 1, Theorem 1, and in some theoretical discussion after Theorem 1. Please note that some terms are introduced just as abbreviations of longer mathematical expressions and thus do not have meanings. Please also note that it is quite difficult to explain some mathematical expressions in words. The added/modified explanations are highlighted in blue color in the PDF with track changes (as in other added parts). We believe that the added explanations will greatly help readers understand the proofs. However, proofs are mathematical concepts and thus some mathematical backgrounds are needed to understand these.

Comment. 4. Improving figure captions:
– Figure captions should clearly explain WHAT is shown on the figure. For example, in Fig S2: what do the on/off panels correspond to? These aspects of the figure should be clearly listed rather than inferred from the main text. In contrast, the explanations of what the results on a figure mean need to be in the main text. Please recheck each figure and make sure the caption explains what you show.

Response. We have worked over all figure legends in the main manuscript (Figs. 1-4) and the Supplementary Information (S1-5 Figs) to improve the understanding. The specific remark about Fig S2: We have explained the meaning of ON and OFF and hope the explanation is sufficient without referring to the main text.

> **Endothelial cell (EC) behavior of atypical behavior inducing microenvironments.** In the original study [14], the authors identified 32 microenvironments predicted to induce atypical EC behavior. EC behavior is interpreted by the signature of four markers (*AKT1*, autocrine *JAG1*, *DLL4a*, and *NRP1*). A microenvironment induces atypical EC behavior if the EC marker signature does not correspond to phalanx, stalk, or tip, or if their signature is not stable in the detected attractors. We based our initial guesses required for attractor analysis on the information provided in the original study, from where we can assign node values for 16 nodes comprising the microenvironment, and 10 nodes with fixed values. For the remaining 116 out of 142 nodes, we tested two scenarios, one in which all remaining nodes are set to 1 (ON scenario), and one where all remaining nodes are set to 0 (OFF scenario). Shown are the EC behaviors interpreted from the detected attractors from our analysis. Rows: Microenvironment numbers (numbers 36-67 corresponding to the original numbering) predicted to induce atypical EC behavior. *ON* column: Results from scenarios where the unkown node values in the initial guess were set to 1 (ON). *OFF* column: Results from scenarios where the unkown node values in the initial guess were set

> to 0 (OFF). Rows (microenvironments) which resulted in attractors corresponding to only one EC behavior (numbers 36-38, and 48-59) showed an instable EC marker signature, and are regarded to induce atypical behavior.

Comment. 5. Improving the description of prior models:
– I have found the introduction of the example networks confusing, especially with respect to why these models are good case-studies for an algorithm with a priory knowledge. In addition, I often felt that these sections do not stand on their own, they expect the reader to be intimately familiar with the two papers the models were published in.

Response. We thank the reviewer for the detailed and constructive feedback. We divided the points into smaller sections to show the point-by-point correspondence with our revision. We have added two new sections "Angiogenesis network" and "Cell cycle control network" to the "Introduction", where we introduce the biological background of the two models and hope our explanations suffice to understand the models.

Some things to consider in order to improve this for BOTH models:
* How did the authors of the original study find their attractors, what did they miss and why?

Response. *Angiogenesis:*
We added the following explanation how the original attractors were found:

> In their study [14], the authors propose a BN which accounts for the regulatory network triggering angiogenesis by inducing specialized EC behavior depending on the microenvironment. They first propose a full BN with $n = 142$ nodes, and reduce it to $n = 64$ nodes. The authors use the reduced model to identify attractors applying an adaptation of a SAT-based approach [17] provided in the BoolNet package [27], interpret which of these found attractors correspond to which EC behavior, and trace the microenvironment that triggered each of these behaviors.

and

> The authors deemed the original network with $n = 142$ nodes too large for attractor analysis, hence, the attractors of the full network remain unknown. This makes the full network an excellent target to demonstrate that our proposed algorithm can easily be applied to a network of this size. Furthermore, our analysis enables us to compare the results from two networks which have been proposed to contain the same information.

Cell cycle:

> In the original study, the authors did not perform an attractor search. Instead, they started from a trivial initial condition where all components were true and present in their neutral (unmodified/unbound) states. From this highly artificial initial state, the authors simulated the model in the absence of *Nutrients* (one of the requirements for the G_1/S transition and a model input) until the model found its natural initial state - corresponding to G0 arrest. This indeed lead to a biologically relevant point

attractor, that could be released into a biologically relevant periodic attractor by setting *Nutrients* to 1 (active), but the authors did not scan for other possible and possibly relevant attractors. This previously detected periodic attractor corresponds to the wildtype. From this wildtype attractor components were systematically and manually removed to represent a set of selected mutant genotypes. However, it remains unknown to what extent other attractors exist. To the knowledge of the authors, a biological bBM of the size of the cell cycle control network has not yet been analyzed using a semi-automatic approach to study the attractor landscape. Hence, the cell cycle control network is a good example to demonstrate the feasibility of our proposed algorithm to the analysis of large-scale BNs, as well as to explore if attractors other than the known ones exist.

* Most important: what, precisely, is the prior knowledge you use, and why do you expert it to improve on their results (why should this part of the state space you single out for priority search lead to novel / more relevant cycles?)

Response. The *a priori* probability is added to the (partially) known attractors presented in the papers. This does not necessarily constitute a good starting point to find novel cycles, but an excellent starting point to determine if the attractors are robust or that if minor perturbations would lead to different attractors.
In the first case study (*Angiogenesis* network), we do indeed identify novel behaviour that was not identified using only the attractor from the reduced system.
In the second case (*Cell cycle control* network), we also identify a number of mutational phenotypes - both those that break the attractor (inviable) and those that still allow normal cell cycle progression (viable). In both cases, the algorithm identifies novel behaviour in "adjacent" parts of the state space.
We added the following explanation about *a priori* information.

The *a priori* information reflects the expected state (0 or 1) of each node and the confidence in this state (a probability $0 \leq p \leq 1$). The first information can be inferred from binarization of experimental data, *e.g.* by assigning the value 1 to an expressed gene. We refer to this information as initial guess. The second part, the probability, is much more difficult to infer from experimental data. However, the beauty of the method is that this value can be tried out empirically: The higher the confidence, the later the value will be switched by the algorithm. Of course, there are also situations when the value can be inferred from data: *E.g.*, the gene can be detected (over a certain threshold) in 7 out of 10 time points: $p_{true} = 0.70$.

Angiogenesis: We added the following explanation to the "Materials and methods" subsection "BN analysis":

Angiogenesis network analysis We consider the originally proposed full angiogenesis BN [14] consisting of $n = 142$ nodes for our analysis. Previously, a reduced version with $n = 64$ nodes was considered for analysis in the original study [14]. Our analysis requires an initial guess and *a priori* probabilities which we base as

much as possible on the original study. In the original study, 16 nodes were identified to comprise the microenvironment (*VEGFC Dp*, *VEGFAxxxP*, *ANG1*, *Oxygen*, *ShearStress*, *JAGp*, *DLL4p*, *WNT5a*, *WNT7a*, *FGF*, *IGF*, *BMP9*, *BMP10*, *TGFB1*, *VEGFC D*, *AMPATP*). In total, 67 patterns of activation reflecting 67 different microenvironments were identified, which we used for our analysis. Furthermore, 10 nodes with a fixed value were used (*ACVR2A*, *BMPRII*, *TGFBRII*, *sGC*, *SMAD4*, *γ-Secretase*, *ADAM10/17* with value 1; *Ryk (sFRP1)*, *DKK1/3*, *BTrCP* with value 0). We assigned an *a priori* probability of 1 to each of these initial guesses.
For the remaining 116 nodes, we tested two scenarios: The ON scenario, in which all remaining nodes are set to 1 in the initial guesses, and the OFF scenario, in which all remaining nodes are set to 0 in the initial guesses. These nodes were assigned the same *a priori* probability, where we tested in total four values (0.7, 0.8, 0.9, and 1.0) for each of the two scenarios. Hence, for each of the 67 microenvironments, the two scenarios (ON and OFF) were tested with four different *a priori* probabilities, adding up to in total 536 tests.

Cell cycle:
We added the following explanation to the "Materials and methods" subsection "BN analysis":

> As initial guess we used an informed guess, a single state from a previously known attractor with period 186 corresponding to the wildtype. The bBM uses *Nutrients* and *Histones* as an input, and can be perturbed with in total four cell cycle inhibitors. The corresponding nodes in the initial guesses were set to 1 and 0, respectively, and their *a priori* probabilities set to 1, except for the scenarios in which the cell cycle inhibitors were explicitly tested.

* How / when does this scenario generalize to other models?

Response. We show how to analyze two Boolean models with different scope, size, and modeling formalism and expect that our algorithm can be applied to a variety of Boolean models.
We use a (partially) previously known attractor, and we assign *a priori* information to this attractor, and *de facto* search in the adjacent state space: Starting from such a prior, we will always find the input attractor, but also possibly unknown attractors with different behaviours and implications - as in those two cases. This "stability analysis" should be generalisable to all Boolean models, as *per* above.
The challenge is when there is no known starting point, as shown by the random starting points in the *Cell cycle control* network (S5 Fig). Here, we do not find any relevant attractors, which likely - in this case - depends on the high frequency of introducing mutations that are - especially in combination - lethal. However, it also shows the value of having prior knowledge, which is what this algorithm is all about.
Hence, the challenge lies in finding this prior knowledge. We expect this to be possible from experimental data, and that even a limited knowledge will be useful. The algorithm compensates for errors and uncertainties in this estimate, as it explores the state space around this initial guess until the user defined CPU time is exhausted.

– Specifically explain / clarify the following:
* Angiogenesis model: "induces typical behavior if all detected attractors correspond to a single

EC behavior" – what does this mean and is this the "typical" behavior (it would help to know what the 16 inputs are, and to make the biology of this model a bit more clear.

Response. We have added a new "Angiogenesis network" section to the "Introduction" where we clarify these points. We specifically added:

> First, we study a BN formulated in the classical way describing the effect of several microenvironments on the behavior of endothelial cells (ECs) during angiogenesis [14]. During angiogenesis, new blood vessels are formed by sprouting from existing ones, a process occurring during embryonic development but also tumor growth. Angiogenesis requires differentiation of ECs, constituting the innermost layer in a blood vessel, into one of three specialized behaviors: phalanx, stalk, and tip. Angiogenesis and EC specialization may be triggered by a hypoxic microenvironment [33].

and

> The authors define in total 16 nodes of the network which comprise the microenvironment. EC behavior is interpreted by the activity configuration of the four marker nodes *AKT1*, autocrine *JAG1*, *DLL4a*, and *NRP1*. A microenvironment induces typical behavior if all detected attractors show an EC marker configuration corresponding to a single and stable EC signature. Otherwise, the microenvironment is regarded as inducing atypical behavior. For example, if the EC marker signature does not correspond to any of the three behaviors or oscillates between them, the induced behavior is regarded as atypical.

We now explicitly mention the 16 nodes which comprise the microenvironemnt in the "Materials and methods" subsection "BN analysis":

> In the original study, 16 nodes were identified to comprise the microenvironment (*VEGFC Dp*, *VEGFAxxxP*, *ANG1*, *Oxygen*, *ShearStress*, *JAGp*, *DLL4p*, *WNT5a*, *WNT7a*, *FGF*, *IGF*, *BMP9*, *BMP10*, *TGFB1*, *VEGFC D*, *AMPATP*). In total, 67 patterns of activation reflecting 67 different microenvironments were identified, which we used for our analysis. Furthermore, 10 nodes with a fixed value were used (*ACVR2A*, *BMPRII*, *TGFBRII*, *sGC*, *SMAD4*, *γ-Secretase*, *ADAM10/17* with value 1; *Ryk (sFRP1)*, *DKK1/3*, *BTrCP* with value 0). We assigned an *a priori* probability of 1 to each of these initial guesses.

* Angiogenesis model: "We could identify periodic 375 attractors in the angiogenesis BN with 142 nodes using a semi-informed initial guess." Please explain your " semi-informed initial guess" before presenting the results of your algorithm.
– Which nodes are set to 0/1 with what probably, and why?

Response. We now explain "semi-informed initial guess" as well as which nodes are set to 0 or 1 in the "Effect of microenvironment on EC behavior revisited (BN with $n = 142$ nodes)" section. It now reads:

Here, we investigated the attractor landscape in the full model with $n = 142$ nodes as provided by the authors [14]. We use the same definition as the authors to interpret EC behavior based on the signature of the four EC markers, and use the same microenvironments inducing a specific behavior. (*I.e.*, microenvironment numbers 1-2, 3-16, and 17-35 induce phalanx, stalk, and tip, respectively; numbers 36-67 induce atypical behavior). Furthermore, our analysis requires initial guesses for each microenvironment, and *a priori* probabilities. We base our initial guesses on the in total 67 microenvironment configurations by the authors of the original study [14], which each comprise 16 nodes. The authors also include 10 nodes with constitutive activity, which we also incorporate in our initial guess. Hence, in total, we can assign 26 node values to our initial guess based on the original study, and assume that their *a priori* probabiltiy is 1 (Materials and methods). Since the initial values of all of the remaining 116 nodes are not reported in the original study [14], we test two scenarios: one in which they are set to 0 (OFF scenario), and one in which they are set to 1 (ON scenario). We call this type of guess with partially known, and partially unknown initial values a semi-informed guess. For these remaining nodes, we test for each of these scenarios (ON and OFF) four arbitrarily chosen *a priori* probabilities: 0.7, 0.8, 0.9, and 1 (Materials and methods).

The "Materials and methods" subsection "BN analysis" now reads:

Our analysis requires an initial guess and *a priori* probabilities which we base as much as possible on the original study. In the original study, 16 nodes were identified to comprise the microenvironment (*VEGFC Dp*, *VEGFAxxxP*, *ANG1*, *Oxygen*, *ShearStress*, *JAGp*, *DLL4p*, *WNT5a*, *WNT7a*, *FGF*, *IGF*, *BMP9*, *BMP10*, *TGFB1*, *VEGFC D*, *AMPATP*). In total, 67 patterns of activation reflecting 67 different microenvironments were identified, which we used for our analysis. Furthermore, 10 nodes with a fixed value were used (*ACVR2A*, *BMPRII*, *TGFBRII*, *sGC*, *SMAD4*, γ-*Secretase*, *ADAM10/17* with value 1; *Ryk (sFRP1)*, *DKK1/3*, *BTrCP* with value 0). We assigned an *a priori* probability of 1 to each of these initial guesses.
For the remaining 116 nodes, we tested two scenarios: The ON scenario, in which all remaining nodes are set to 1 in the initial guesses, and the OFF scenario, in which all remaining nodes are set to 0 in the initial guesses. These nodes were assigned the same *a priori* probability, where we tested in total four values (0.7, 0.8, 0.9, and 1.0) for each of the two scenarios. Hence, for each of the 67 microenvironments, the two scenarios (ON and OFF) were tested with four different *a priori* probabilities, adding up to in total 536 tests.

* Cell cycle model: what is a DESIRED attractor that stops your algorithm's search?

Response. The algorithm stopped after a user specified CPU time, and the distinct attractors were evaluated. We now added the following sentence:

All attractor searches were conducted so that the **ATTapriori** algorithm was run until a CPU time limit was exceeded.

* Cell cycle model: ”then analyzed the periodic attractors (S1 Table) and found that one corresponds to the wildtype, 39 correspond to viable deletion mutants”
– What do you mean by this? which model are you analyzing, the WT or the mutant one? If the WT, how and why would some of its attractor correspond to viable deletion mutants? If you run both, why would you report the attractors in aggregate?

Response. For our analysis, the model was always the same, we only used different initial guesses. However, in the bipartite Boolean modeling logic, removal of certain combination of states is equivalent to removing the component itself. To clarify this approach, we now explain in the section ”Analysis of a cell cycle control network (bBM with $n = 3158$ nodes)”:

> First, we studied the attractors of the genotype corresponding to the wildtype phenotype where we used the previously known periodic attractor as initial guess. We regard this an informed guess, where we also set two necessary network inputs *Nutrients, Histones* to 1, and four other inputs, corresponding to chemical inhibitors of the cell cycle, to 0. We tested four different, arbitrarily chosen *a priori* probabilities, 0.7, 0.8, 0.9, and 1 in different combinations, except for the in total six inputs, where the *a priori* probabilities are all set to 1 (see Materials and methods). We discovered 66 unique periodic attractors with period 186, and 125 unique point attractors (Fig 4). We then analyzed these 66 unique periodic attractors (S1 Table) by manually scanning for nodes that are constitutively 0, and compare this to the wildtype attractor. If the constitutively inactive states all belong to one gene, we consider this gene to be absent in the attractor. We found that one attractor corresponds to the wildtype, 39 correspond to viable deletion mutants (gene-specific sites at The Saccharomyces Genome Database (SGD) [30]), two correspond to deletion mutants with conflicting reported phenotypes (PP2A, Net1), and 16 to deletion of hypothetical kinases and phosphatases introduced in the model construction process (not testable). Only one corresponds to a lethal deletion mutant; *sec2*, where the essential function is not part of the model. The remaining 7 are technical artefacts, where an infeasible pattern of macroscopic states was active in the initial perturbation. Hence, all the newly discovered periodic attractors correspond either to deletion mutants or to technical artefacts, and no new basins of attractions in the wildtype were discovered. The 125 unique point attractors (S2 Table) can by definition only correspond to lethal phenotypes. We manually examined the attractors for inactive nodes, and assigned lethal mutations if the inactive nodes corresponded to one or several genes. We found that 71 of the 125 detected point attractors indeed correspond to known lethal mutants. 28 mutants have a viable phenotype *in vivo*, but redundancy mechanisms that compensate for loss of their functions are not included in the model. 18 mutants showed a missing structural component, and hence, the phenotype is lethal (model phenomenon, technical artefact). The remaining eight attractors constitute technical and other mutants which could not be clearly categorized, or where the reported *in vivo* phenotype is conflicting.

* Cell cycle model: ”an invalid pattern with no biological meaning”
– what does this mean, why invalid, and how is this distinguished form attractors that do have

biological meaning? What is their phenotype? What does it tell you about the model, or a mutant version, that it has these non-biological attractors? (especially since this appears to be different from a ”lethal” phenotype ...; are there supposed to be stable apoptotic attractors in this model, which are biologically relevant, just ”dead”?)

Response. Thank you for helping us clarifying this. This goes back to the bipartite Boolean modeling formalism [28, 29], that we now explain in greater detail in the subsection ”Boolean network” in the ”Introduction”.

> A special form of BNs are bipartite Boolean models (bBMs, simplified example in Fig 1C). While they, like classical BNs, consist of nodes and vertices, the modifications of a single gene can be expressed explicitly and in mechanistic detail. The bipartite network structure has two types of nodes, state nodes, corresponding to elemental states (*i.e.* empirical observables such as specific phosphorylations), and reaction nodes, corresponding to elemental reactions (*i.e.* decontextualized biochemical reaction events). State nodes influence reaction nodes, and hence, all incoming edges of a reaction node describe how a reaction is regulated, allowing a mechanistic description of biological processes (details in [28, 29]).
> The two types of Boolean modeling formalism, classical and bipartite, have different consequences for the perturbation analysis. In a classical BN, a gene or component can be knocked out by disabling the node it corresponds to. In a bBM, most components are represented by multiple nodes corresponding to different states at one or more distinct sites (residues or domains). Also, some, but not all, may be represented as genes, including transcription and translation reactions. However, for many components, the model used here only includes posttranslational modifications and complex formation. At each level, there may be one or more elemental states involved which are all represented by individual nodes. Hence, there is a one-to-many relationship between a gene and the number of nodes which represent the different elemental states of the gene in a bBM. Conversely, if all states corresponding to a single residue or domain are set to false, then the component has *de facto* been deleted as long as no synthesis occurs in the model. This has specifically implications for the interpretation of detected attractors in the cell cycle control network. Our proposed algorithm perturbs the node values based on probabilities, and hence sometimes creates deletion mutants by removing the gene or by removing the only remaining state for a specific site or domain in a protein. In both cases, the modification has effectively altered the genotype of the model. Similarly, an essential component such as RNA polymerase II could be removed, or one of the macroscopic states could be added or removed, leading to a model that is no longer biologically meaningful. The latter is rare, and we consider it a technical artifact. This also explains why a bBM such as the cell cycle control network cannot be simulated with asynchronous updates. Since components are represented by multiple states that get produced or consumed, a reaction may consume (remove) a source state and produce (add) a product state. If the two states are updated independently, a consumption update that is not matched by a production update would eventually result in the removal of the only remaining state of a specific site or domain, and hence, resulting

in the effective deletion of that component. The synchronous updates ensure that this cannot happen.

The explanation of invalid patterns is related to the bipartite Boolean formalism. The explanation now reads:

> Due to the bBM formalism, a few considerations need to be taken for the attractor analysis (see Introduction). The **ATTapriori** algorithm may find attractors in which two or more mutually exclusive states (*e.g.*, DNA both replicating and replicated at the same time) are simultaneously true, or a necessary component is not true. While these attractors are technically possible, they do not convey any biological meaning. We regard these attractors as technical artefacts and refer to them as *invalid*. In addition, the algorithm may remove a gene or the last true state of a specific site, effectively creating a deletion mutant of the corresponding component(s) and hence altering the genotype of the model. Next, we regard all other attractors to correspond to a biologically relevant genotype, with either of two possible corresponding phenotypes: viable or lethal. We regard an attractor to correspond to a viable phenotype if all macroscopic processes are traversed, as this would be required for a cell to successfully duplicate. This is only possible for periodic attractors. Similarly, we regard an attractor to correspond to a lethal phenotype if the attractor is a point attractor, or a periodic attractor which does not traverse all macroscopic states. Such a lethal phenotype may correspond to a mutant in which an essential gene is missing.

– The sentence: "Finally, we used 10 random states as initial guesses, and only detected point 328 attractors, regardless of switching the input Nutrients to on or off" brings up a few issues * clarify what method you used to start from 10 random states, and find 238 point attractors. Was this your ATTapriori algorithm? In that case, what does "10 random states as initial guesses" mean, exactly?

Response. We now explain the random initial states in the following way:

> Finally, we tested random initial states as initial guesses. Our previously known periodic attractor corresponding to the wildtype is an informed guess about an existing periodic and biologically relevant attractor. Here, we used 10 random initial states to test if our algorithm can detect periodic and/or biologically relevant attractors with random, and hence, uninformed guesses. We tested three scenarios, one with completely random initial guesses, one in which the input *Nutrients* was set to on, and one in which *Nutrients* was set to off. In all three scenarios, only point attractors were detected (S5 Fig).

And in the "Materials and methods" section:

> The random initial guesses were created by assigning values of 0 or 1 to all nodes in the network following a binomial distribution.

* Why 10 initial conditions? how does that compare to other attempts used by you or the authors of the original study?

Response. Our algorithm does not solve the problem of finding an inital cyclic guess. The random initial states were a test to check if an initial state could be obtained in a feasible way. To our knowledge, a similar test has not been conducted before. However, we must also note that the complete random guess is likely to introduce a number of mutations that prevent cell cycle progression (see above). This could also be an important cause for the lack of success starting from a completely random initial state.

* In my experience, a synchronous Boolean model with a cyclic attractor that is supposed to represent a robust biological rhythm needs to have a pretty large attractor basin leading to that limit cycle. Ideally, the limit cycle or something quite similar to it should show up even under asynchronous update! Does this sentence indicate that the original, published biological limit cycle itself is very difficult to find without prior knowledge? If yes, this questions the validity of the original model as a robust cell cycle model; if no, please clarify why not.

Response. We relate this question to the underlying bipartite Boolean network structure and the consequences for finding attractors and interpretation of these attractors.
We added a new section "Boolean Network" to the introduction and updated Figure 1 which now also contains a panel C and D. We now explicitly explain bipartite Boolean models:

> A special form of BNs are bipartite Boolean models (bBMs, simplified example in Fig 1C). While they, like classical BNs, consist of nodes and vertices, the modifications of a single gene can be expressed explicitly and in mechanistic detail. The bipartite network structure has two types of nodes, state nodes, corresponding to elemental states (*i.e.* empirical observables such as specific phosphorylations), and reaction nodes, corresponding to elemental reactions (*i.e.* decontextualized biochemical reaction events). State nodes influence reaction nodes, and hence, all incoming edges of a reaction node describe how a reaction is regulated, allowing a mechanistic description of biological processes (details in [28, 29]).
> The two types of Boolean modeling formalism, classical and bipartite, have different consequences for the perturbation analysis. In a classical BN, a gene or component can be knocked out by disabling the node it corresponds to. In a bBM, most components are represented by multiple nodes corresponding to different states at one or more distinct sites (residues or domains). Also, some, but not all, may be represented as genes, including transcription and translation reactions. However, for many components, the model used here only includes posttranslational modifications and complex formation. At each level, there may be one or more elemental states involved which are all represented by individual nodes. Hence, there is a one-to-many relationship between a gene and the number of nodes which represent the different elemental states of the gene in a bBM. Conversely, if all states corresponding to a single residue or domain are set to false, then the component has *de facto* been deleted as long as no synthesis occurs in the model. This has specifically implications for the interpretation of detected attractors in the cell cycle control network.

– Please explain your work with mutant genotypes and phenotypes more clearly, especially: "detected mutational genotypes with a directly interpretable corresponding phenotype based on the type of detected attractor. That is, the algorithm automatically performed an in silico

mutagenesis screen, a task which had to be performed manually previously."

Response. The consequences for detecting attractors in bipartite Boolean networks addressing the comment about invalid patterns now reads:

> Our proposed algorithm perturbs the node values based on probabilities, and hence sometimes creates deletion mutants by removing the gene or by removing the only remaining state for a specific site or domain in a protein. In both cases, the modification has effectively altered the genotype of the model. Similarly, an essential component such as RNA polymerase II could be removed, or one of the macroscopic states could be added or removed, leading to a model that is no longer biologically meaningful. The latter is rare, and we consider it a technical artifact. This also explains why a bBM such as the cell cycle control network cannot be simulated with asynchronous updates. Since components are represented by multiple states that get produced or consumed, a reaction may consume (remove) a source state and produce (add) a product state. If the two states are updated independently, a consumption update that is not matched by a production update would eventually result in the removal of the only remaining state of a specific site or domain, and hence, resulting in the effective deletion of that component. The synchronous updates ensure that this cannot happen.

We now explain the interpretation of the found attractors in the "Analysis of a cell cycle control network (bBM with $n = 3158$ nodes)" in the "Results":

> Due to the bBM formalism, a few considerations need to be taken for the attractor analysis (see Introduction). The **ATTapriori** algorithm may find attractors in which two or more mutually exclusive states (*e.g.*, DNA both replicating and replicated at the same time) are simultaneously true, or a necessary component is not true. While these attractors are technically possible, they do not convey any biological meaning. We regard these attractors as technical artefacts and refer to them as *invalid*. In addition, the algorithm may remove a gene or the last true state of a specific site, effectively creating a deletion mutant of the corresponding component(s) and hence altering the genotype of the model. Next, we regard all other attractors to correspond to a biologically relevant genotype, with either of two possible corresponding phenotypes: viable or lethal. We regard an attractor to correspond to a viable phenotype if all macroscopic processes are traversed, as this would be required for a cell to successfully duplicate. This is only possible for periodic attractors. Similarly, we regard an attractor to correspond to a lethal phenotype if the attractor is a point attractor, or a periodic attractor which does not traverse all macroscopic states. Such a lethal phenotype may correspond to a mutant in which an essential gene is missing.

* You appear to claim that the algorithm finds mutant genotypes automatically. If you mean genotypes – how? Mutant phenotypes require a change in the network structure or logic rules; in what way does your algorithm do this, how does it test the phenotype of these mutant models, and how dies it check if it matches known biology (or predicts a mutant phenotype, if

it is yet untested)? How and why do you claim an automatic mutant screen?

Response. This question is related to the bipartite Boolean modeling formalism, where components are represented by their states. If all states corresponding to a specific residue or domain in a component are false, then the component is considered to be absent. Hence, removing the last state of a specific residue *de facto* equals a deletion.

> We discovered 66 unique periodic attractors with period 186, and 125 unique point attractors (Fig 4). We then analyzed these 66 unique periodic attractors (S1 Table) by manually scanning for nodes that are constitutively 0, and compare this to the wildtype attractor. If the constitutively inactive states all belong to one gene, we consider this gene to be absent in the attractor. We found that one attractor corresponds to the wildtype, 39 correspond to viable deletion mutants (gene-specific sites at The Saccharomyces Genome Database (SGD) [30]), two correspond to deletion mutants with conflicting reported phenotypes (PP2A, Net1), and 16 to deletion of hypothetical kinases and phosphatases introduced in the model construction process (not testable). Only one corresponds to a lethal deletion mutant; *sec2*, where the essential function is not part of the model. The remaining 7 are technical artefacts, where an infeasible pattern of macroscopic states was active in the initial perturbation. Hence, all the newly discovered periodic attractors correspond either to deletion mutants or to technical artefacts, and no new basins of attractions in the wildtype were discovered. The 125 unique point attractors (S2 Table) can by definition only correspond to lethal phenotypes. We manually examined the attractors for inactive nodes, and assigned lethal mutations if the inactive nodes corresponded to one or several genes. We found that 71 of the 125 detected point attractors indeed correspond to known lethal mutants. 28 mutants have a viable phenotype *in vivo*, but redundancy mechanisms that compensate for loss of their functions are not included in the model. 18 mutants showed a missing structural component, and hence, the phenotype is lethal (model phenomenon, technical artefact). The remaining eight attractors constitute technical and other mutants which could not be clearly categorized, or where the reported *in vivo* phenotype is conflicting.

* How do you interpret the mutant phenotype?

Response. In the cyclic attractors found with our algorithm, we scan for states that are always false and compare this to the wild type attractor. This rapidly identifies the missing components. In the point mutants, this is more tedious, requiring manual analysis. Our starting point was to look for states that were false only in a few attractors - as the deletions are expected to be rare. We now explain in the "Results" section:

> We discovered 66 unique periodic attractors with period 186, and 125 unique point attractors (Fig 4). We then analyzed these 66 unique periodic attractors (S1 Table) by manually scanning for nodes that are constitutively 0, and compare this to the wildtype attractor. If the constitutively inactive states all belong to one gene, we consider this gene to be absent in the attractor.

and

> The 125 unique point attractors (S2 Table) can by definition only correspond to lethal phenotypes. We manually examined the attractors for inactive nodes, and assigned lethal mutations if the inactive nodes corresponded to one or several genes.

We also added the following explanation to the "Materials and methods" section:

> The cell cycle control bBM [26] describes the control and regulation of three macroscopic cellular processes required for cell cycle progression: DNA replication, spindle pole body duplication, and cell growth. In the model, each macroscopic process traverses a set of irreversible biological states, which allows monitoring of the cell state and cell cycle progression. ...
>
> ...The proposed algorithm perturbs the initial guess. Due to the architecture of the bBM, this does not only perturb the starting vector, but also has a chance of permanently removing one or more components, *de facto* creating a deletion mutant. Hence, a small fraction of the detected attractors corresponds to technical artefacts, *e.g.* if none or several of the mutually exclusive macroscopic cell cycle states were initiated as true. These artefacts were omitted from the following analysis. We evaluated if a newly detected attractor corresponds to a viable phenotype by checking i) if at each time point, exactly one macroscopic state is true in each macroscopic process, ii) if each macroscopic process traverses through all its states. From this follows that only periodic attractors potentially correspond to a viable phenotype. Point attractors may correspond to a lethal phenotype induced by one or a combinations of lethal mutations. This happens if all complementary states for a particular site are set to 0 (inactive) for an essential component whose turnover (synthesis) is not considered in the model (see Introduction), *de facto* creating a deletion mutant. Similarly, if a macroscopic state is turned on or off, it is very likely to introduce an unfeasible state where none or more than one of these mutually exclusive states are 1 (active).

Comment. I hope you find these comments helpful and constructive. I think your work deserves attention form anyone wrangling a network larger than 25 nodes (so, many of us), and thus a convincing paper that is easy to read can help.

Response. Thank you for your constructive comments. We revised the paper with taking all comments from you and other reviewers into account.

2 Response to Reviewer 2

Comment. In this manuscript the authors study the problem of finding specific attractors of Boolean networks when information about the 0/1 patterns are known with certain probability p. A formula in terms of p is derived that shows the expected number of trials to find such attractor. It is shown that if p>0.5 then the expected time to find an attractor is lower than the expected time using an exhaustive-search approach.
To the best of my knowledge this idea is new and the results are valid. I also tested the submitted codes with one of the examples and it seems to work well. However, there are some questions/concerns that need to be addressed.

Response. We thank you for your valuable comments. We revised the manuscript according to your comments as detailed below.

Comment.
============================
About the algorithm/implementation
============================
1. It seems that the time complexity is for finding ONE attractor only. What can be said about finding ALL attractors for which there is a priori information? There seem to be something about that in the manuscript, but it is not clear.

Response. Since prior knowledge gives a hint for only one attractor, it does not help to reduce the time complexity for finding ALL attractors. We have added the following sentences. Please note that, to the best of our knowledge, there does not exist any known algorithm that can enumerate ALL attractors with guaranteed worst-case or expected time complexity better than $O(2^n)$.

> It is to be noted that the algorithm can be modified so that it can enumerate all attractors by removing "If the attractor is a desired one, output it and exit." of STEP 3 and merging the identical attractors. However, it would take more than $O(2^n)$ time (because it will examine all 2^n starting vectors) and thus the resulting algorithm is meaningless. Since the purpose of this algorithm is to make use of *a priori* information on some global state in a specific attractor, it is reasonable that the algorithm is not useful for enumerating all attractors.

Comment. 2. The Github site is empty. The code should be there since the manuscript states "The ATTapriori algorithm is implemented in C, and the source code is available at https://github.com/takutsu5/ ."

Response. Sorry for the mistake. We had put the code in some sub-directory (because we were not familiar with GitHub). After receiving a message from the editor, we put the code in the top directly.

Comment. 3. It is strange that the code internally uses
load_net("network.boolnet");
load_init_val("init_vals.txt");

instead of just reading from user input when the compiled code is used. This means that a user has to edit and compile the source code every single time to able to use it for a different example. It would be easier if the code is compiled once and then used as

```
./AttPrior network.boolnet init_vals.txt >attlog2\\
```

or similar. Is there a reason for the current implementation that requires modifying the source code?

Response. In the code provided in GitHub, the code works as you suggested.

Comment.
================
About the examples.
================
4. [This is a major concern] It is not clear what was the advantage in using the proposed algorithm in the examples. Was it faster? Were there new insights that would not be found without the algorithm? I suppose it is the former. If so, the authors should explain (in percentage, perhaps), how much faster the algorithm was compared to an exhaustive search approach. Perhaps show that a specific attractor was found X% faster with the proposed algorithm? The examples have to clearly illustrate why this algorithm was better than an exhaustive search approach at least for one attractor.

Response. In order to clear the advantages of our method, we have performed additional experiments to show how few state transitions our method can reach the target attractor compared to a random searching method. As a result, for the random N-K networks of sizes $N = 10, 20, 30$, and 40 and $K = 2$ with the prior probability $= 0.90$, the average of total lengths of trajectories of our method were shortened to about 1/40 at the maximum. This result means that our method can find the target attractor more quickly than the random search. This is because the search for the target attractor can be started from a good initial state by *a priori* information. Therefore, we have added the following sentence.

> Finally, we compared the total lengths of trajectories until reaching a target attractor with (**ATTapriori**) and without (**Random**) *a priori* information. In this experiment, a target attractor was set in advance, which was detected by *BoolNet* package of R. The **Random** method starts from a random initial state and repeats state transitions until an attractor is found. If the found attractor is the target attractor, it outputs the total lengths of trajectories and terminates. Otherwise, it starts from another random initial state and repeats the above procedure. On the other hand, in **ATTapriori**, the same procedure was done except that the initial states are decided by **ATTapriori** algorithm. The Fig 2C shows that the average total lengths of trajectories of 100 experiments not including period lengths until reaching the target attractors for the sizes of $N = 10, 20, 30$, and 40, where the prior probability in **ATTapriori** was set to 0.90. From the results, the average total length of trajectories of **ATTapriori** were shortened to about 1/40 at the maximum.

3 Response to Reviewer 3

Comment. This paper proposes to use prior knowledge to accelerate the identification of attractors in Boolean networks. It uses prior probabilities as an heuristic to reduce the expected number of states to explore before finding the first attractor. Such prior knowledge is usually not available but it could correspond to expected behaviours based on experimental observations or hints obtained from other, less complex, models. The authors discuss the use of their approach on random networks and on two large models, along with some new biological insight. Overall the topic of the identification of attractors is important and the proposed method makes sense in some cases. I did not fully check and the analysis of the expected complexity, but the general principle seems valid, but be based on some assumptions which are not stated very clearly. However I find some of the claims presented here a bit excessive.

Response. We thank you for your valuable comments. We revised the manuscript according to your comments as detailed below.

Comment. The current state of the art approaches rely on constraint solving. In practice constraint solvers explore many branches and it is indeed true that for some very peculiar models they would have to explore the whole state space. However actual models enable to cut large parts of the search space: for example if a component A is required for the activation of a component B, then branches with A fixed at 0 and B fixed at 1 are not explored, not just because they are less likely to contain a solution, but because it can structurally not contain one. The expected complexity is in practice much better than O(n$\hat{2}$). Several methods exist for stable states (using prolog, SAT solvers, explicit decision diagrams or ASP), as well as the approximations of complex attractors through stable motifs (also called trap spaces or symbolic steady states, which are missing from the paper). Note that using these approaches, the set of constraints could be extended to restrict the search to attractors matching some expected patterns, and the absence of matching attractor could then be formally verified without exploring all states. However this involves some knowledge about the inner working of these methods.

Response. We understand that many practical methods reply on constraint solving which cuts large parts of the search space. Indeed, $O(1.587^n)$ time and $O(1.985^n)$ time algorithms in [21, 22] were developed based on this idea (and using an existing theoretically efficient algorithm for SAT, which also relies on cutting of the search space). However, these algorithms can only handle attractors with period 1 [21] and period 2 [22] for restricted subclasses of BNs. We also understand that many methods based on constraint solving are practically very useful. However, the main issue is that none of existing methods theoretically guarantees the worst-case or expected time complexity better than $O(2^n)$ for reasonably large subclasses of BNs. In order to clarify this point, we added the following sentences along with some new references).

> These algorithms are based on cutting of unnecessary partial states. For example, suppose that note y is activated only if node x is activated. Then, we need not examine states including $(x, y) = (0, 1)$. However, for example, if an XOR function is assigned to y, this strategy does not work. The divide and conquer approach was also employed in [22], which shows that detection of a periodic attractor of a constant period can be done in polynomial time if the maximum degree is bounded

by a constant and a given network is decomposable into small pieces (precisely, the treewidth of a given network is bounded by a constant). However, in some cases, BNs may not be decomposed into small pieces. For example, if a given BN is a clique (all nodes are connected by edges), there is no way to decompose it.

It is worthy to mention that many practically efficient methods have been developed for detection and/or enumeration of attractors using such techniques as logic programming [2], SAT solvers [17], binary decision diagrams [1], and answer set programming [3], as well as representation/approximation of complex attractors through stable motifs [4], symbolic steady states [5], and trap spaces [18]. Although these methods are practically very useful, there is no theoretical guarantee better than $O(2^n)$ on either worst-case or expected time complexity.

Comment. In the introduction, the authors argue that no method have a worst case complexity better than O(n2̂) (i.e. enumerating all states). This is true. Then they claim to break this limit using prior knowledge. Here they compare their theoretical EXPECTED complexity to the theoretical WORST case of other approaches. Then they only search for a SOME attractor while other approaches target the formal identification of ALL of them. As the method relies on enumeration plus simulation, it's theoretical worst case complexity is still O(n2̂). Note that in deterministic models, finding at least one attractor assuming that it has a short length has always been a fairly easy problem: pick some initial state (random or not) and perform a simulation with a rotating memory until recovering the last memorized state (with some ways to avoid looping forever if the memory is too short). This work seems to mainly propose the use of prior knowledge as heuristic for the initial sampling of initial states. It can be useful for models too large for a more formal analysis, but it is limited to deterministic models (finding attractors through simulation in the (more realistic) non-deterministic case is much harder) and gives less informative results.

Response. It seems that many practical methods works better than in $O(2^n)$ expected time. However, to our knowledge, no method has a theoretical proof that its expected time complexity is better than $O(2^n)$. Therefore, as mentioned above, we have added the following sentence.

> Although these methods are practically very useful, there is no theoretical guarantee better than $O(2^n)$ on either worst-case or expected time complexity.

We understand that it is easy to find some attractor by using your suggested approach, and our proposed method works only on deterministic BNs. We also understand that the worst-case time complexity of the method is not better than $O(2^n)$. Therefore, we have added the following sentences in Introduction Section and Discussion Section, respectively.

> It is worthy to note that if it is enough to find some attractor, there exists a trivial approach: start from a random initial state and then follow the trajectory until reaching an attractor. This method works efficient unless the trajectory is very long. However, it cannot control the type of a detected attractor, even for singleton or periodic. Therefore, *a priori* information would be useful to control the search towards detection of the desired attractor. It should also be noted that our algorithm examines 2^n initial states in the worst-case (i.e., *a priori* information is not at all useful) and thus does not improve the worst-case time complexity.

> Although we have considered synchronous BNs in this work, various models of asynchronous BNs have been proposed and studied [1, 3]. Unfortunately, our approach cannot be applied to such BNs because trajectories are not uniquely determined. Therefore, it is interesting to study how *a priori* information can be utilized for attractor detection in asynchronous BNs.

Comment. The description of the sampling method could be improved, especially as the expected complexity is evaluated on the number of trials. The text suggests that this corresponds to the number of tested initial state but this does not make sense. Is it the length of the trajectory leading to the first state which is part of an attractor? The described algorithm has no specified memory size and limit on the length of explored trajectories, meaning that it could explore the whole state space on the first initial state and still end up finding an attractor thus be counted as success. If the expectation is that the selected initial state is itself part of the attractor, then the memory size and the cost of comparison are small, but it needs a limit on the length to decide that it failed. Furthermore, the examples in the explanation use probable states of 0, thus the optimized sampling order is an order which could well be used by default in absence of prior information.

Response. The analyses in Lemma 1 and Theorem 1 only discuss whether or not the initial state is included in a desired attractor, and do not depend on the length of the trajectories. Therefore, Lemma 1 and Theorem 1 are valid. The time complexity of the whole procedure is discussed after Theorem 1 (in both the original version and this revised version), which depends on the length of the trajectories, where the length of the trajectory is the sum of the length of a path reaching an attractor and the length (i.e., period) of that attractor. Since considering exponentially long attractors is not realistic, we assumed the length of the desired attractor is polynomially bounded and thus the discussion on the time complexity is reasonable. In order to clarify these points, we have added the following sentences.

> Even if the trajectory is not bounded by a polynomial, we can modify the algorithm so that each trial is finished if the length of the trajectory exceeds some given steps (some polynomial steps). Since the proofs of Lemma 1 and Theorem 1 only discuss whether a given initial state is the same as the target state, this modification does not affect these theoretical results. It may affect the correctness of the original algorithm (i.e, the original algorithm may miss the desired attractor) because the period of the desired attractor may not be bounded by a specified polynomial, where the period of an attractor corresponds to a cyclic part of the trajectory (not the whole part of the trajectory). However, it is reasonable not to consider very long (e.g., exponentially long) attractors because any usual organism cannot live for exponentially long periods. For example, suppose that 1 step corresponds to 1 micro second (i.e., 10^{-6} second). Then, even for $n = 100$, 2^n is greater than 4×10^{16} years. Limiting the length of the trajectory is also useful to reduce the space complexity. The algorithm need to memorize all states in the trajectory in order to find a cycle, which implies that the worst-case space complexity would be $O(2^n \cdot n)$ because the length of the trajectory can be $O(2^n)$ in the worst case. However, if we limit the length of the trajectory by some constant and it is enough to find one desired attractor, the space complexity would be $O(n)$.

Comment. Last, the paper mentions a check that the found attractor is "a desired one" without really saying what this means. Is it a comparison to the probabilities used as input? What would be the error margin for accepting it? Is this score computed during the simulation and used to decide when to interrupt it?

Response. We are sorry that we have not given a formal description about a desired attractor in theoretical analyses. We have added the following sentences before Lemma 1.

> Suppose that $x_i = 0$ holds with probability p for all $i = 1, \ldots, n$ in some specific global state $\mathbf{x}_t$ of the target attractor, where $p > 0.5$. Note that if $x_i = 1$ has the probability p ($p > 0.5$) it is enough to exchange the roles of 0 and 1 for such nodes. In theoretical analyses, the objective is to give an upper bound of the number of trials until the algorithm examines $\mathbf{x}_t$ as a starting vector. Therefore, we need to modify STEP 3 as follows:
>
> > If $\mathbf{x}_0 = \mathbf{x}_t$, output it and exit.
>
> Of course, this modified algorithm is meaningless in practice because it is impossible to know $\mathbf{x}_t$ in advance. However, the expected number of trials (i.e., the expected number of tested $\mathbf{x}_0$s) for this modified algorithm gives an upper bound of that for the original algorithm because it examines the whole trajectory beginning from $\mathbf{x}_0$ (much more than one global state $\mathbf{x}_0$ per trial).

In practice, there is no formal or definite way to define "a desired attractor". This point was discussed in the last part of "Time complexity analysis" Section. In order to clarify this point, we have newly added the following sentences just below the pseudocode.

> Note that how to decide whether the current attractor is a desired one is not a trivial task. If we know some exact criteria (e.g., allowable range of the attractor period and/or states of specific nodes), we can add a subroutine to check it. Otherwise, the algorithm can be terminated if the number of trials exceeds a specified number or CPU time exceeds the time limit. Then, we may manually check (maybe with some user-customized computer program to rule out obviously non-desired ones) whether or not there exists a desired one among the attractors reported so far.

Comment. The paper contains the following statement: "most practical Boolean functions can be evaluated in polynomial time and the lengths of trajectories in most practical BNs are considered to be not very long". If this is true then a simulation from any initial state would lead to an attractor in a short time. THis is often true, but not a formal guarantee. I suspect that the advantage of the proposed approach is to shorten the trajectory leading to an attractor by selecting a good initial state.

Response. We believe that the assumption on Boolean functions is reasonable. Otherwise, even simple simulation would not be possible. As for the lengths of the trajectories, our assumption may be too strong. However, as explained before, theoretical analyses in Lemma 1 and Theorem 1 do not depend of the length of the trajectory. Furthermore, as explained before, we can limit the search length of the trajectory, and a simulation from any initial state would lead to some attractor but may lead to a non-desired one. Therefore, the advantage of our approach is to start

the search from multiple states that are expected to be contained in a desired attractor. Here, we report that the lengths of the trajectories are not so long by a simple simulation. We measured the average lengths of trajectories of 100 experiments from random initial states to attractors using random N-K networks and the angiogenesis network as a real network. As a result, the average length of trajectories is less than only 300 even when the size of random network is 100. Furthermore, the average length of trajectories of the angiogenesis network is much shorter than that of random N-K network of size 100 even though its size is 142. Therefore, in some practical BNs, the lengths of trajectories to reach attractors are not considered to be very long. We have added the following sentences.

> Second, we performed another experiment to show the distribution of the lengths of trajectories until reaching attractors. The Fig 2B indicates the average length of trajectories of 100 experiments from random initial states to attractors without period lengths for random N-K networks whose sizes are $N = 5, 10, 20, 50, 100$ and indegrees are $K = 2$. As a real network, the lengths of trajectories of the angiogenesis network of size $N = 142$ was also measured (its details are described in the next section) [14]. The symbol '$*$' indicates the averages of period lengths. Although the average lengths of trajectories becomes longer as the size gets larger, it is less than 300 even when the size of network is 100. In addition, the average length of trajectories of the angiogenesis network is much shorter than that of random networks of size $N = 100$.

Comment. I feel that a more extensive comparison of the total number of explored states until the discovery of the first attractor with and without prior knowledge (and using bad prior knowledge) is needed to evaluate the effect of prior knowledge (unless this is what figure 2 is supposed to show?).

Response. As mentioned in **Response** to **Reviewer 2**, we have performed additional experiments to show how few state transitions our method can reach the target attractor compared to a random searching method. As a result, for the random N-K networks of $N = 10, 20, 30,$ and 40 and $K = 2$ with the prior probability $= 0.90$, the average total lengths of trajectories (i.e., the total number of explored states) of our method were shortened to about 1/40 at the maximum. We have added this result as Fig 2C and the following sentences.

> Finally, we compared the total lengths of trajectories until reaching a target attractor with (**ATTapriori**) and without (**Random**) *a priori* information. In this experiment, a target attractor was set in advance, which was detected by *BoolNet* package of R. The **Random** method starts from a random initial state and repeats state transitions until an attractor is found. If the found attractor is the target attractor, it outputs the total lengths of trajectories and terminates. Otherwise, it starts from another random initial state and repeats the above procedure. On the other hand, in **ATTapriori**, the same procedure was done except that the initial states are decided by **ATTapriori** algorithm. The Fig 2C shows that the average total lengths of trajectories of 100 experiments not including period lengths until reaching the target attractors for the sizes of $N = 10, 20, 30,$ and 40, where the prior probability in **ATTapriori** was set to 0.90. From the results, the average total length of trajectories of **ATTapriori** were shortened to about 1/40 at the maximum.

Comment. During the analysis on random networks, the use of ”perfect” prior knowledge based on a state identified to be part of the attractor is a very strong assumption. Some noise should be added to it so that the most probable state would not be exactly part of the attractor. This may be related to this sentence that I did not understand: ”The 0/1 values of each node of the detected attractors were changed into 1/0 with the reverse given a priori probability in order to generate an initial state of the random N-K networks”.

Response. Since we cannot have perfect knowledge on the desired attractor (otherwise, nothing should be done), we need some procedure (subroutine) or some way to check whether a a given attractor is a desired one. This point was given in a previous response. As for the latter part of the comment, sorry for the confusing sentence “The 0/1 values of each node of the detected attractors were changed into 1/0 with the reverse given a priori probability in order to generate an initial state of the random N-K networks”. We revised the sentence as follows.

> In order to avoid matching with the target attractors in the first trials, the bit vectors generated by changing each bit of the target attractors according to the prior probability are given as the initial states.

Comment. The authors illustrate their approach on two models which they claim are untractable with existing tools. I did some tests using the original model of endothelial cells (with 142 components, it is much smaller than the cell cycle model). Using bioLQM it was possible to identify all 1’097’952 attractors of this model within 4 seconds on a laptop (using decision diagrams, note that printing the result takes significantly longer). Within 20s, I could identify the first 100’000 trap spaces of the same model using PyBoolNet (using an ASP solver). With it I could also pick a random initial state, identify the 45 components which are structurally fixed in this state and use this to find the 2 potentially reachable trap spaces (both fixed points) within half a second. I also tested this model with BNS (using a SAT solver to identify synchronous attractors) which provided hundreds of results within one second. The author used a modified version where all components with fixed values have been turned into input components. This strongly increases the total number of attractors but probably does not affect the difficulty of finding at least one deterministic attractor with AttPrior. With this variant, the analysis of all fixed points becomes hard, but the analytical identification of some attractor, or their full identification after setting the initial state remain easy. Considering these results, I would say that this model remains perfectly tractable using existing tools. Despite the large number of attractors, finding all potential attractors for any specific initial state is fairly easy even in the non-deterministic case.

Response. Thank you for your comment. We agree that a Boolean network with $n = 142$ nodes can be analyzed using existing methods. We added the following motivation why we chose this model for analysis:

> The authors deemed the original network with $n = 142$ nodes too large for attractor analysis, hence, the attractors of the full network remain unknown. This makes the full network an excellent target to demonstrate that our proposed algorithm can easily be applied to a network of this size. Furthermore, our analysis enables us to compare the results from two networks which have been proposed to contain the same information.

Comment. The second example model is much much harder to work with: loading it into bioLQM or PyBoolNet is a challenge as these tools use heavy representations for the Boolean rules. Using some personal code with lighter data structures, I could find quickly some attractors by sampling deterministic simulations without having to use prior knowledge, but it involved fairly long traces. Turning the full model into a "good" set of constraints was much much slower than finding some solutions for the set afterwards. In this case, I would indeed say that formal analysis becomes problematic and that the proposed approach is useful. I suspect that rewriting the model to replace complex rules with additional components would facilitate formal analysis, but this is not an easy task either. From my (biased) perspective, this highlights the need for improving the translation of Boolean rules into sets of constraints. The proposed approach should be used for very large models until this happens (if ever).

Response. We agree with the remark that the algorithm should be used for large Boolean models. However, we are not aware of any other larger or more challenging Boolean models to test than the ones we analyzed here. We added the following motivation why we chose the second network:

> To the knowledge of the authors, a biological bBM of the size of the cell cycle control network has not yet been analyzed using a semi-automatic approach to study the attractor landscape. Hence, the cell cycle control network is a good example to demonstrate the feasibility of our proposed algorithm to the analysis of large-scale BNs, as well as to explore if attractors others than the known ones exist.

Taken together, our algorithm can be conveniently used to identify attractors of different scope, size, and formalism (classical and bipartite). We hope our algorithm enables other researchers to develop and analyze larger Boolean networks in the future.

Comment. While testing the provided code on these two models I ran into a few problems: * the github repository was empty until very recently, and this version does not work with the first model * the code provided in the supplementary archive did work but needs to be compiled to change the input files (which is solved in the github version). * while it uses the bnet format (good point for avoiding the use of yet another new format), it does not properly parse it: components with fixed values, header line and comments should be handled to be able to use existing models

The code would be useful after improving the support for the bnet format and including some examples in the github repository. A git tag to find the content of the supplementary from the repository would be nice.

Response. We have added a revised code (attprior2.c) to the GitHub repository, which can handle fixed values, header line, and comments. We have also added two additional examples (toy_model_2, toy_model_3).
However, we did not modify the codes in the supplementary archive because these were adjusted for specific purposes.
For the additional experiments, new codes (length.r, length_weinstein.r, and total_length.r) and a network model (2017_Weinstein_fullMod_sorted.boolnet) have been added to the supplementary archive (comp_exp.zip). The details of them can be found in README.txt in the archive.
